# Sensitive Terahertz Detection and Imaging Driven by the Photothermoelectric Effect in Ultrashort‐Channel Black Phosphorus Devices

**DOI:** 10.1002/advs.201902699

**Published:** 2020-01-19

**Authors:** Wanlong Guo, Zhuo Dong, Yijun Xu, Changlong Liu, Dacheng Wei, Libo Zhang, Xinyao Shi, Cheng Guo, Huang Xu, Gang Chen, Lin Wang, Kai Zhang, Xiaoshuang Chen, Wei Lu

**Affiliations:** ^1^ State Key Laboratory of Infrared Physics Shanghai Institute of Technical Physics Chinese Academy of Sciences 500 Yu‐Tian Road Shanghai 200083 China; ^2^ University of Chinese Academy of Sciences No. 19A Yuquan Road Beijing 100049 China; ^3^ School of Physical Science and Technology ShanghaiTech University Shanghai 201210 China; ^4^ CAS Key Laboratory of Nano‐Bio Interface and Key Laboratory of Nanodevices and Applications i‐Lab Suzhou Institute of Nano‐Tech and Nano‐Bionics (SINANO) Chinese Academy of Sciences Ruoshui Road 398 Suzhou Jiangsu 215123 China; ^5^ School of Nano Technology and Nano Bionics University of Science and Technology of China Jinzhai Road 96 Hefei Anhui 230026 China; ^6^ Zhejiang Lab Artificial Intelligence Town No.1818 Wenyixi Road Hangzhou 311100 China; ^7^ Institute of Molecular Materials and Devices Department of Material Sciences and Department of Macromolecular Sciences Fudan University Shanghai 200433 China

**Keywords:** black phosphorus, imaging, photothermoelectric effect, terahertz detectors

## Abstract

Terahertz (THz) photon detection is of particular appealing for myriad applications, but it still lags behind efficient manipulation with electronics and photonics due to the lack of a suitable principle satisfying both high sensitivity and fast response at room temperature. Here, a new strategy is proposed to overcome these limitations by exploring the photothermoelectric (PTE) effect in an ultrashort (down to 30 nm) channel with black phosphorus as a photoactive material. The preferential flow of hot carriers is enabled by the asymmetric Cr/Au and Ti/Au metallization with the titled‐angle evaporation technique. Most intriguingly, orders of magnitude field‐enhancement beyond the skin‐depth limit and photon absorption across a broadband frequency can be achieved. The PTE detector has excellent sensitivity of 297 V W^−1^, noise equivalent power less than 58 pW/Hz^0.5^, and response time below 0.8 ms, which is superior to other thermal‐based detectors at room temperature. A rigorous comparison with existing THz detectors, together with verification by further optical‐pumping and imaging experiments, substantiates the importance of the localized field effect in the skin‐depth limit. The results allow solid understanding on the role of PTE effect played in the THz photoresponse, opening up new opportunities for developing highly sensitive THz detectors for addressing targeted applications.

## Graphical abstract

A new strategy is proposed to apply the photothermoelectric (PTE) effect in an ultrashort channel using the titled‐angle evaporation technique. Such a detector exhibits tremendous field‐enhancement and high‐efficiency THz photon absorption across a broadband frequency range beyond the skin‐depth limit, introducing an avenue for deeply understanding the role of the PTE effect and targeted applications for scalable, fast imaging.

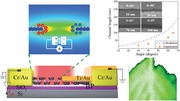

Terahertz (THz) wave, the lastly unexplored electromagnetic radiation that is defined as the photon frequency between 0.1 and 10 THz, has recently received unprecedented interests for a wide range of applications in fields of medicine, security, and noninvasive quality testing that are either complementary to or simply cannot be achieved at other electromagnetic radiation frequency bands.^1^, ^2^, ^3^, ^4^ However, the progress of THz technologies is strongly hindered by the lack of fast, sensitive photodetectors as well as portable powerful‐source for high‐performing system operating at room temperature.^2^, ^5^

Up to now, peoples have devoted a great deal of efforts to develop compact solid‐state sources and detectors, and successive breakthroughs have been made recently due to the progress of interdisciplinary study between material science, terahertz photonics, and nanofabrication technologies, allowing for the feasibility of engineering devices and arrayable photodetectors with ad hoc properties to target the expected performance and functionalities.^6^, ^7^, ^8^, ^9^, ^10^ 2D materials offer extensive playground for studying the quantum state of matter that can exhibit intriguing electronic and optoelectronic properties such as ultrafast charge transport as well as tunable photon absorption.^6^, ^7^, ^8^, ^9^, ^11^, ^12^, ^13^ Application of graphene for THz detection has ushered in the new era of exploitation of 2D materials for bridging the gap between microwave electronics and infrared photonics with different physical mechanisms such as photothermoelectric (PTE), plasma wave, and bolometric effects reported to date.^14^, ^15^, ^16^, ^17^, ^18^, ^19^, ^20^

The quest for alternative material in 2D family has been the driving force of current research endeavor in the aim of expanding application capability at THz frequency. Even though layered transition metal dichalcogenides (TMDCs) show exceptional optoelectronic properties, their large bandgap, low mobility, and large resistance make the THz photodetection difficult to be achieved owing to the very limited photon absorption.^21^, ^22^, ^23^, ^24^ Graphene is a usable photothermoelectric material, featuring larger Seebeck coefficient, high carrier mobility, and air stability. However, this material suffers from weak light absorption efficiency due to the structure of monolayer/bilayer and large dark current, resulting in the low responsivity and high noise equivalent power (NEP). In addition, graphene is not available of polarization‐sensitive detection because of isotropic band structure.^18^ The appearance of black phosphorus (BP) offers a good trade‐off between graphene and TMDCs. It possesses a small direct bandgap of 0.3 eV with graphite‐like structure, endowed with hole mobility well above 1000 cm^2^ V^−1^ s^−1^, allowing for high‐speed operation and huge carrier density tunability.^25^ In contrast to graphene, the puckered hexagonal structure of BP generates an inherent anisotropy and peculiar large Seebeck coefficient.^26^ For all these reasons, BP can overtake the limit factor of TMDCs and graphene, bringing undisputed benefits in the viewpoint of THz detection for high speed and high sensitivity. During the past couple of years, there are several experimental demonstrations of few‐layer BP‐based photodetection in the THz range, typically in the overdamped self‐mixing regime in a nanometer field‐effect transistor. Noise equivalent power less than 40 nW Hz^−0.5^ has been reached, comparable with that of best bilayer graphene‐based ones.^27^

Similar to the graphene counterpart, the photothermoelectric effect in BP is also electron‐heat‐driven photoresponse through intraband processes at THz frequency if asymmetry such as asymmetrical doping or antenna feeding is presented. Due to its in‐plane anisotropy, BP exhibits both high electrical and thermal conductivities. It has been both theoretically predicted and experimentally verified that BP has large Seebeck coefficient around 198 µV K^−1^.^28^, ^29^ Nevertheless, photothermoelectric effect in BP has not been fully exploited at both infrared and THz bands.

The main obstacle to exploit the PTE effect for THz detection is the tremendous mismatch between the wavelength of the incident radiation and the relatively small photoactive area, resulting in a low responsivity.^30^ Engineering the efficient way at a low cost for hot carriers production and preferentially photocurrent is crucial to meet the requirement of an ideal detector. Plasmonic nanostructure exhibits extraordinary light transmission and greatly enhanced localized field and these superiorities have been successfully utilized in the fields of nanophotonics, bio‐chemical sensing and photodetection at infrared or visible part of spectrum on basis of self‐organized growth and high precision nanofabrication of nanopatterns.^17^, ^20^, ^31^, ^32^, ^33^, ^34^ However, delocalization of electric field happens to the plasmonic nanostructure at THz band. Although it has been reported that spoof surface plasmons on perforated structure with period are close to the level of THz band, it is low efficiency and incommensurate with the photoactive area of 2D atomic layer.^35^ To maximize the coupling efficiency, it has been reported that a large modulation depth in graphene can be achieved by placing photoactive material at micrometer gap of subwavelength aperture, resulting in both improved absorption and increased bandwidth.^14^ The featured size for such electric or magnetic properties is much smaller than the wavelength, but nonetheless much larger than the skin depth and it has been shown that the field enhancement keeps increasing by decreasing the slit width on basis of expensive nanolithography technique.^36^ Here, we solve this issue by introducing ultrashort channel between the two‐sleeves of bow‐tie antenna by using a facile method, where the incident light is strongly concentrated on the photoactive area of the multilayer BP. As a consequence, the hot electrons can be extensively generated at the slit, known as “hot spots.” In this design, we can achieve several advantages of BP photothermoelectric THz detector. First of all, strong enhancement of electric field can be made at channel length smaller than skin depth. Second, fast extraction of hot electrons is feasible across the ultrashort channel composed of asymmetrical metal–semiconductor interfaces. Moreover, it has the advantage of broadband capability applicable to other 2D materials for imaging system with both low power consumption and high sensitivity. The PTE detectors reported here can achieve a maximum responsivity larger than 297 V W^−1^ and minimum optical NEP less than 58 pW Hz^−0.5^.

In the following, we will first explain the field enhancement concept in the slit down to the skin‐depth limit and how the PTE detector of our design works, followed by a complete photoelectric characterization. Then, quantitative comparison of device response under different infrared pumping is devoted to probe the PTE effect experimentally. It has also demonstrated that optimized design of antenna structure and manipulation of Seebeck coefficients allow for further improvement of the proposed PTE detector.

To realize our terahertz detector with ultrashort channel, asymmetrical electrodes with dissimilar metals are achieved by using a tilted‐angle evaporation technique,^37^, ^38^ as shown in **Figure** **1** (see also Experimental Section). Here, multilayer BP is exfoliated from BP single crystal (Section S2, Supporting Information) onto a high resistive Si substrate (ρ ≈ 20 000 Ω cm) which is covered by a 300 nm SiO_2_ in Figure 1a. The 30 nm thick flakes are identified and characterized by both the Raman and atomic force microscopy (AFM) in Figure 1f,i. Then, the BP flake is contacted with two sleeves of bow‐tie antenna made from 10 nm/60 nm (Cr/Au) metallic stack with 4 µm gap by using ultraviolet lithography, electron beam deposition, and lift‐off processes in Figure 1b. In order to concentrate the incoming THz radiation into a very small spot area, a second lithography is devoted to open a window on the channel, followed by electron beam deposition along different tilted angles θ as shown in Figure 1c. Since the generation of photocurrent from hot carriers requires a gradient in the Seebeck coefficient, a sequential deposition of 10 nm/60 nm (Ti/Au) film is devoted following the second lithography. Because the device is placed at a tilted angle in respect to that of the metal deposition, the pre‐existing Cr/Au electrode leaves a shadow behind for the latter deposition, which ultimately leave a sub‐100 nm channel in a facile and controllable method as shown in Figure 1c. Finally, an atomic layer deposition (ALD) process is carried out to deposit a 20 nm thick Al_2_O_3_ layer as a protective layer to avoid BP degradation as shown in Figure 1d. By changing the evaporation angle θ, the length of channel can be varied from 100 nm down to 20 nm in a controllable manner. Figure 1g displays the photoactive region of BP, with the two sleeves of antenna serving also as electrodes for electrical readout. Figure 1g,h depicts the microscopy of one of the finished the devices with 30 nm channel mainly discussed in this work. Micro‐Raman spectrum and current–voltage characteristic are shown in Figure 1i,j. SEM images of different channel derived by changing titled angle θ are shown in Figure 1k, where it can be found that the measured channel lengths approach approximately well with the calculated ones (blue dashed line) according to the relationship *L* = *h* × tan(θ), where *L*, *h*, and θ are channel length, electrode thickness (70 nm in this work), and evaporation angle, respectively. The discrepancy from experimental data is reasonable as the flake thickness is not considered.

**Figure 1:**
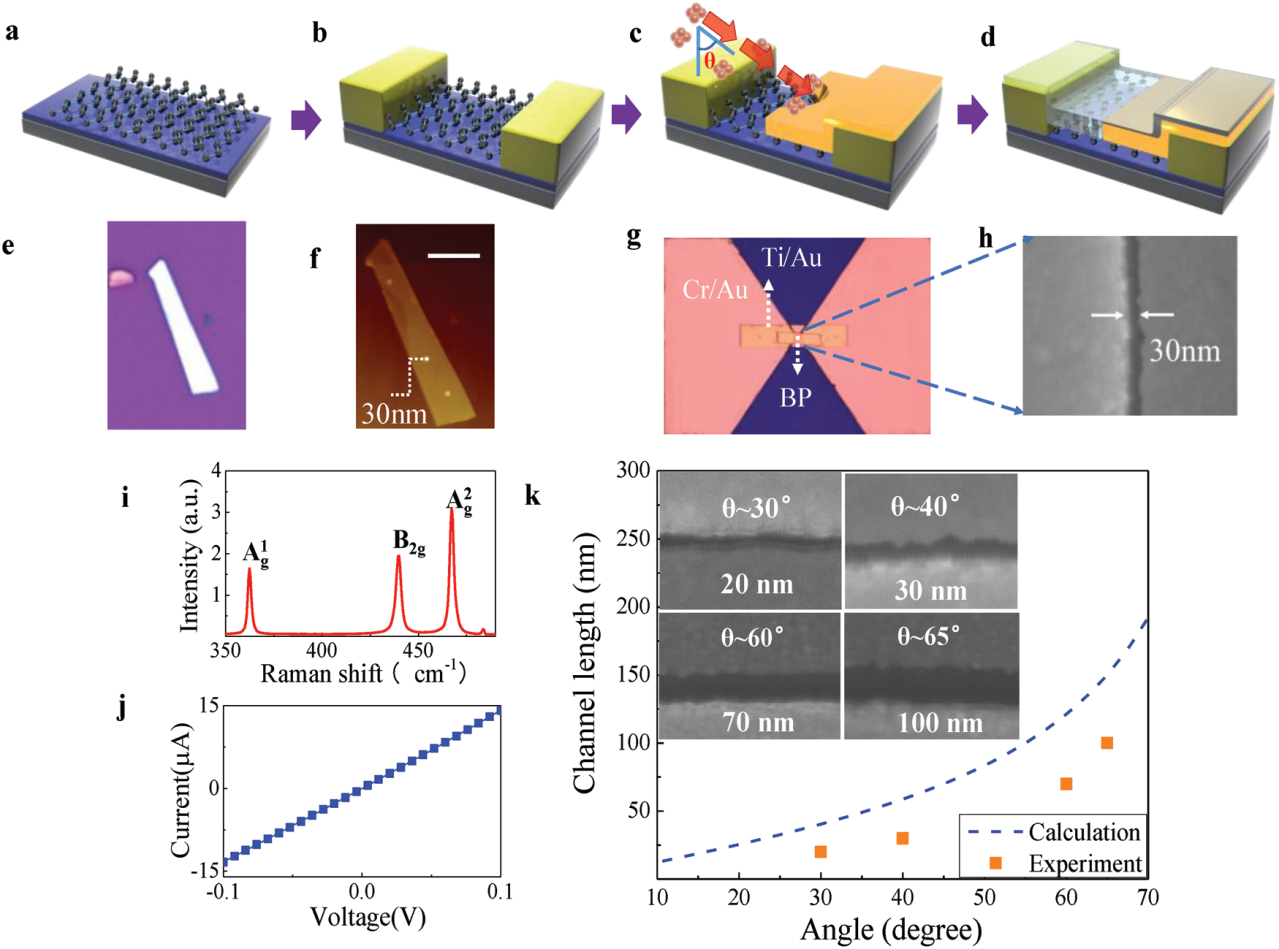
Ultrashort channel PTE detector fabrication by the tilted‐angle technique. a–d) Schematic of the fabrication process: (a) and (b) show the BP device contacted in advance by symmetrical electrodes with 4 µm long channel via ultraviolet lithography and lift‐off processes. (c) and (d) display the sub‐100 nm channel and asymmetrical contacting formation in a BP photodetector by tilted‐angle disposition and lift‐off process after secondary ultraviolet lithography. (e) and (f) are the optical microscopy and AFM image of the BP flake for device patterning. (g) shows the microscopy of one finished device with 30 nm channel length with central part enlarged in (h) (SEM, scanning electron microscopy). i,j) The Raman spectrum and current–voltage characteristic of the 30 nm device. (k) shows the dependence of channel length on the titled angle θ, dashed line is the result from theoretical prediction.

The fact that the simultaneously usage of antenna for concentrating light and electrical connection has the advantage that hot spot (strong THz focusing) and photoactive region can be reasonably overlapped. Qualitatively, when THz wave impinges onto the two sleeves of antenna at normal incidence, ac current is induced on the surface, resulting in charges accumulation at the edges of the antenna near the channel.^36^ When the slit (or channel) in the center of the antenna continues to decrease toward the deep sub‐wavelength and below, the charge density at the edges increases accordingly, leading to the giant enhancement of electric field as depicted in **Figure** **2**a. To show quantitatively the in‐plane overlap between the maximum field amplitude and photoactive region, finite difference time domain (FDTD) simulation is performed for a frequency range of 0.02–0.5 THz (wavelength of 15–0.6 mm) with perfect conductor approximation of metallic plate. We find that the presence of the ultrashort channel profoundly modifies the terahertz field amplitude giving rise to a hot spot like profile at the channel (inset of Figure 2b). The area‐normalized amplitude, which indicates the terahertz field enhancement, increases as the channel decreases due to the increased accumulation of induction charges at the two edges. The enhancement can be larger than two orders of magnitude at 0.29 THz when the channel length is 30 nm, indicating an electromagnetic compression ratio over 10^4^.

**Figure 2:**
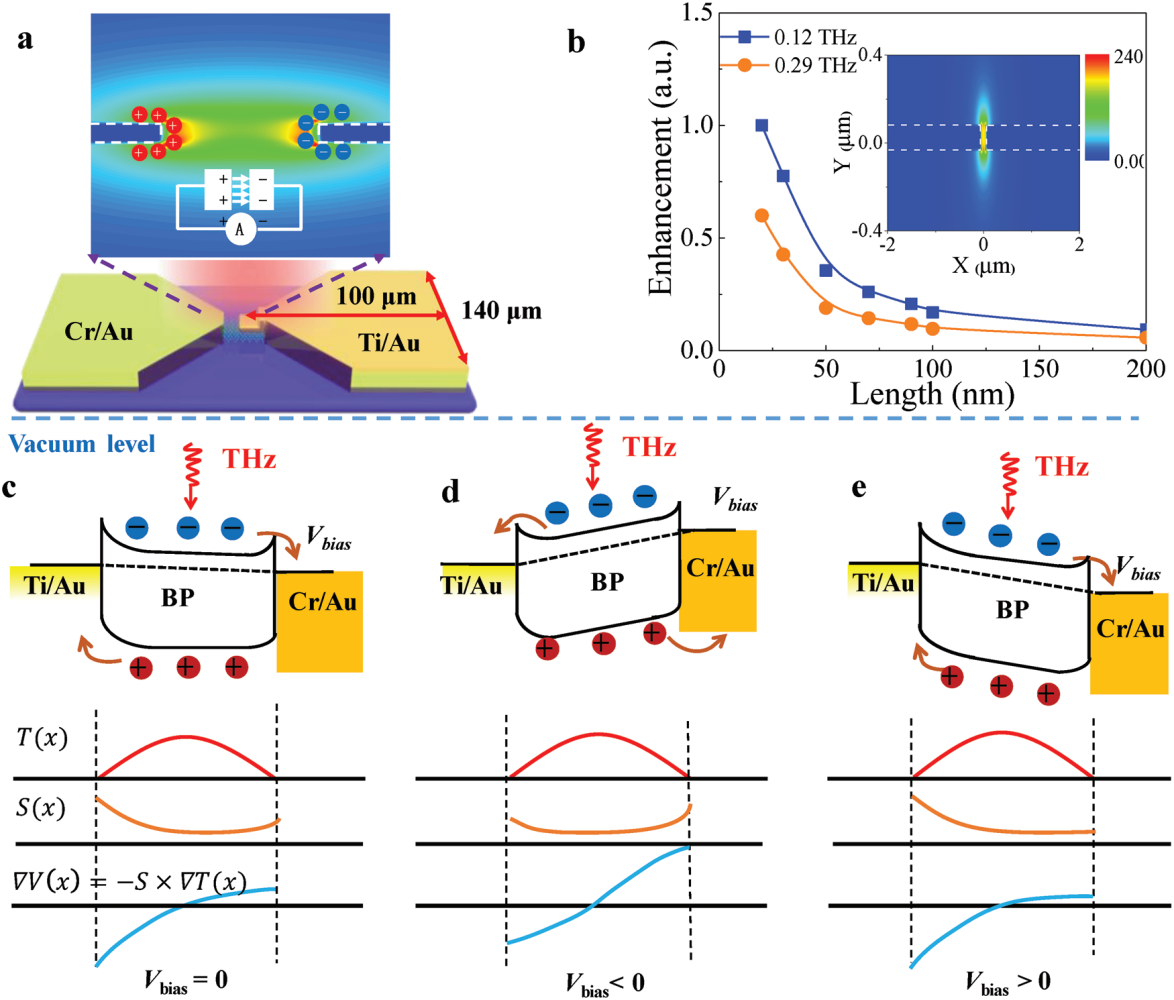
a,b), Terahertz field enhancement concept and simulation results: a) Light‐induced alternating current charges the sub‐100 nm slit, resulting in the localized electric‐field enhancement as shown in the gradual color contour. b) The cross‐section of the antenna (white dotted line) and the THz field distribution near the sub‐100 nm channel at 0.12 and 0.29 THz used in this work, showing that about two orders of magnitude enhancement at the hot spot. Inset shows the profile of “hot spot” in the sub‐100 nm slit between the two metallic contacts. c–e) Illustration of the band diagram at the junction regions of sub‐100 nm channel formed by two dissimilar metallic contacts as shown schematically in Figure 1, and profiles across the device of electron temperature T(*x*), Seebeck coefficient S(*x*), and potential gradient ∇*V*(*x*)*= −S ×* ∇*T*(*x*). The photoresponse is proportional to the integral of ∇*V*(*x*) over the length of the device.

Figure 2c–e presents the operational principle of our device: electrons/holes in multilayer BP are heated up by the incident field at the hot spot, and the two contacts serve as heat sinks, resulting in a temperature gradient distribution ∇*T*(*x*) as a function of position *x* along the channel. Because of the dissimilar metal induced‐dopings at two metal–BP interfaces, the Seebeck coefficient are asymmetric across the channel (*S*_L_ ≠ *S*_R_ for the left and right metal–BP interfaces, *S = π^2^k*_B_ *^2^T*/*6eE*_F_).^39^ Diffusion of hot electrons creates a potential gradient ∇*V*(*x*) = −(*S*_L_ − *S*_R_)∇*T*(*x*), and the total signal is the integral of ∇*V*(*x*) over the channel, which is nonzero due to the asymmetry. However, depending on the bias conditions, there exists three regimes of device operation. When *V*_bias_ = 0 V, the net photocurrent can be driven from Cr/Au to Ti/Au as a result of the metallic work function difference (*W*_Cr_ − *W*_Ti_ ≈ 0.3 eV), which means that the holes would be more depleted and Seebeck coefficient is larger at the right contact (Cr/Au)–BP interface.^19^, ^29^, ^39^ However, when a negative bias is applied from Cr/Au to Ti/Au electrodes, the Seebeck coefficient difference would be reduced or even reverse its sign, so that the photocurrent changes its direction.

Following above understandings in both field enhancement and PTE process, we proceed our experiment by quantifying the performance of BP‐based terahertz photodetector with different channel lengths for better comparison. During the characterization, the device is illuminated by a chopped continuous‐wave and the photosignal is recorded by using a lock‐in technique. The terahertz source is with the power intensity of ≈10 µW mm^−2^ (0.24–0.29 THz) and ≈8.6 µW mm^−2^ (0.12 THz) after experiencing multiplication of fundamental frequency from Agilent E8257D (Experimental Section). All the measurements mentioned above are taken under ambient condition at room temperature. To be more clarified, the current–voltage characteristics of studied devices under the same “ON/OFF” modulated radiation are also recorded and presented in Figure S5a (Section S7, Supporting Information).

For better understandings, we will focus on the results of device obtained with 30 nm channel. **Figure** **3**b displays the time resolved photoresponse at zero bias voltage under impingement by different incident photon frequencies. All the pulse shapes are well preserved with good signal‐to‐noise ratio, indicating that our device is suited for multiband detection. Such a broadband capability is in well accordance with the theoretical prediction of the subwavelength bow‐tie structure (Figure 2b). To show how the antenna improves the detector's sensitivity, the device is measured under normal illumination at 0.29 THz with its electric vector along the variable angle in respect to the antenna axis. Figure 3c shows that the responsivity is smallest when the THz electric field is parallel to the slit. Due to the strong focusing of the localized electric field as depicted above, the signal is enhanced by a factor of 120 when the THz electric field is perpendicular to the slit by exciting the ac charge oscillation, which ultimately results in the lobe‐like polarization diagram (Figure 3c).

**Figure 3:**
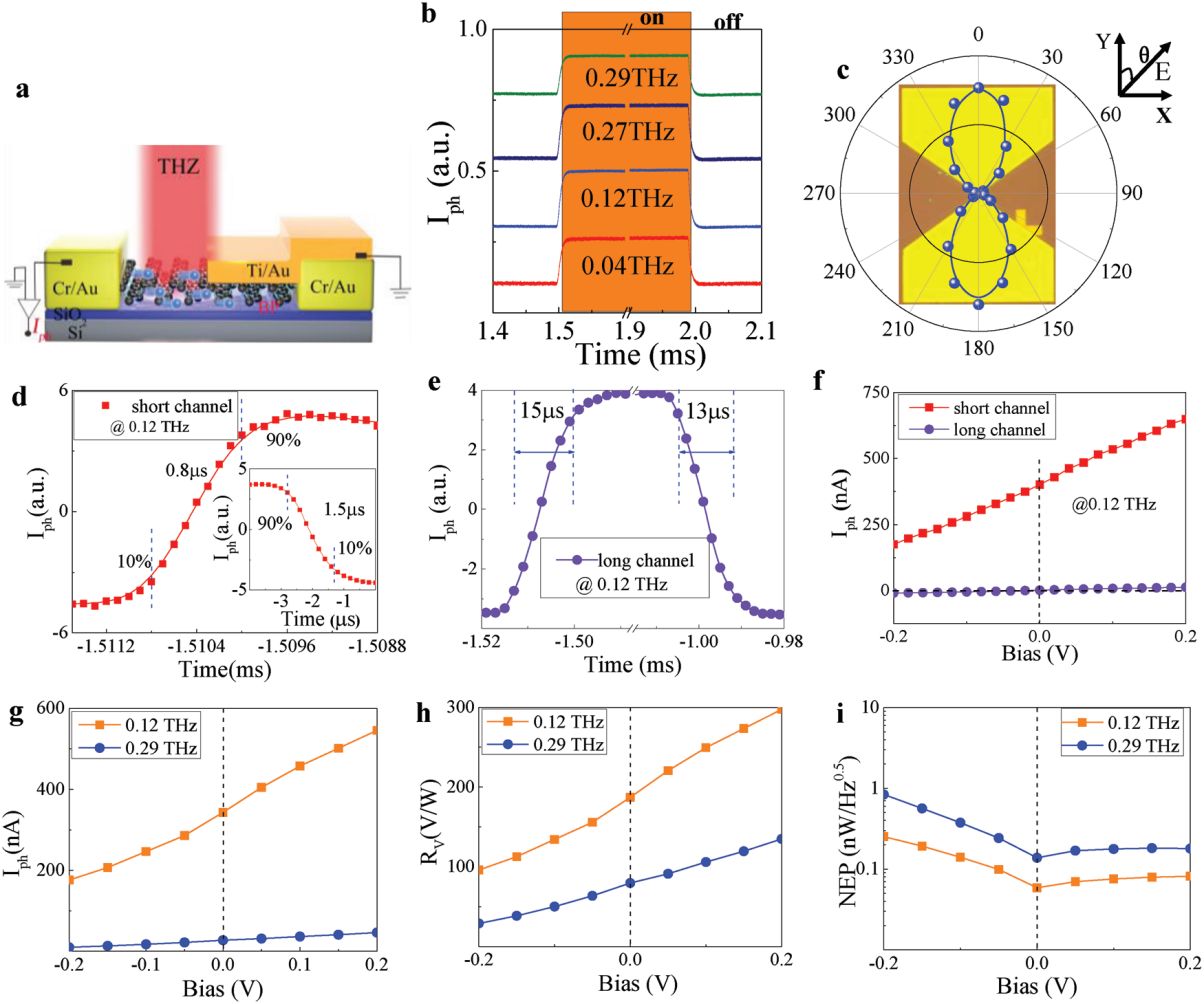
a) Electrical configuration of the PTE device with the terahertz radiation focused by off‐axis parabolic mirrors, here Ti/Au is grounded. b) The pulsed current response at various excitation frequencies, showing broadband nature of PTE effect dominated mechanism. c) Polarization dependence of the 30 nm device at 0.29 THz. d,e) Comparison between response time of the 30 nm and 4 µm devices. f) The bias voltage dependences of the response in the ultrashort channel PTE device and 4 µm channel device at 0.12 THz. g–i) The photocurrent, responsivity, and NEP versus the bias voltage at 0.12 and 0.29 THz, respectively.

In the following, we would like to discuss about the speed of our PTE detector. A single period temporal response is shown in Figure 3d. To extract the 3 dB electrical bandwidth efficiently, the time for the photocurrent to increase from 10% to 90% on the rising edge or analogously on the falling edge of a single pulse is defined as the rise or fall time, respectively. Our PTE detector yields a rise time (τ_r_) of 0.8 µs and fall time (τ_f_) of 1.5 µs at zero bias voltage, substantiating the real‐time imaging capability. In the meantime, the temporal response of 4 µm long device is also presented in Figure 3e and rise/fall time of about 15 µs can be derived. In our setup, the response speed is mainly limited by the bandwidth of current preamplifier (SR570) with 1 MHz full width half maximum (FWHM). Such an impressively short response time is leveraged on the channel length and carrier mobility in black phosphorus. Furthermore, the conceptual design is also applicable to other layered materials system, which is expected to improve the performance in a more aggressively way.

To verify the efficiency of enhanced absorption ability under overlapping between hot spot and photoactive area, the photoresponse is also compared between 30 nm and the 4 µm long ones (without titled angle method) in Figure 3f. Here, we exploit the bias tunability of response as delineated in Figure 2 to identify the role of PTE effect under 0.12 THz radiation source. Without bias, the flow of hot carriers is determined by the work function difference between the two metal–semiconductor interfaces, and the hot holes flow from Cr/Au to Ti/Au due to larger work function and Seebeck coefficient at Cr/BP interface, i.e., *I*_PTE_ ∝ (*S*_R_ − S_L_). By applying a positive voltage with electrical configuration as shown in Figure 3a, the holes flow more easily from Cr/BP interface toward Ti/BP interface, so that the photocurrent can be increased as shown in Figure 3f. While the response is inhibited when the bias is applied along the opposite direction (The same trends can also be observed from the evolution of current–voltage characteristics under THz radiation in Section S7, Supporting Information).

During above process, it is interesting to see that there are significant differences in the bias‐dependent properties after titled angle evaporation. Especially at the zero bias point, there is no photocurrent presented in the long channel device (without asymmetrical contacting), while the PTE photocurrent is predominantly large in our ultrashort channel device, certifying the success in asymmetric metal‐induced doping enabled by the tilted angle method. Furthermore, the photocurrent of PTE device exhibits weaker bias dependence, in well accordance with the above discussions. While the photocurrent of 4 µm long channel detector change its sign between forward and backward bias, behaving like the one relying on the resistive change under THz radiation, but at the expense of magnified noise under bias. Thus, the results show that the PTE effect is responsible for the observed THz response in ultrashort channel device featuring lower noise and power consumption. In addition, similar bias‐dependent behavior can be found at other incident frequencies in Figure 3g due to the broadband nature of the hot electron process. Hitherto, the PTE effect explored here depends on the maximum absorption ability and nonequilibrium between hot carriers and its surroundings, and the frequency depends solely on the structure when strong in‐plane coupling is satisfied. By introducing asymmetry of intrinsic doping, the photocurrent can be preferentially given rise in a convenient way.

We now proceed to quantify the responsivity R of our PTE detectors in Figure 3h, which is defined principally as the ratio of the signal voltage (*V*_ph_ = *I*_ph_·*R*, *R* ≈ 7.3 kΩ in our device) to the incident power onto the device. Since our device is an order of magnitude smaller than the incident wavelength, the diffractive limit area λ^2^/4 is taken as a reference for the incident power (see the Experimental Section). Thus, the voltage responsivity represents a lower limit and can be larger than 297 and 135 V W^−1^ at 0.12 and 0.29 THz, respectively. To our knowledge, it is among the best reported ones based on BP and is competitive with other commercially available ones^15^, ^16^, ^18^, ^27^, ^40^, ^41^, ^42^, ^43^, ^44^ (Table S1, Supporting Information). Higher performance is expectable with more area‐matched antenna in both size and resistance.

From the perspective of practical applications, NEP is another key figure of merit to evaluate the performance of a photodetector, which is defined as the lowest detectable power in 1 Hz bandwidth, and can be expressed as NEP = *v*_n_/*R*_v_, and *v*_n_ is the root mean square of the noise voltage and *R*_v_ is the voltage responsivity of the device. For our devices, the thermal Johnson–Nyquist noise (*v*_t_) that normally dominates the noise for the terahertz detector is included, in addition to the shot noise (*v*_b_) resulting from uncorrelated arrival of the electrons under bias current. We estimate the shot noise from the device bias point where we measure the response. Other noise source such as the flicker noise with system‐specific mechanisms usually takes place at low modulation frequency.^45^, ^46^ As the modulation frequency increases beyond 10 kHz, the 1/*f* noise current of the detector quickly decreases and becomes negligible. In our measurement, the photocurrent remains unchanged even with the modulation frequency increasing up to 10 kHz. The subsequently decreasing of photocurrent is caused by the decreasing of output power from our electronic source (Section S5, Supporting Information). That means, our detector is intrinsically faster without significant decay even at modulation frequency higher than 40 kHz. Inherently, the total noise limit can be expressed in terms of *v*_n_ = (*v*_t_^2^ + *v*_b_^2^)^1/2^ = (4*k*_B_ *Tr* + 2*qI*_d_ *r* ^2^)^1/2^, and *k*_B_ is Boltzmann's constant, *T* is the temperature of the detectors, *r* is the resistance, *q* is the elementary charge, and *I*_d_ is the dark current of the device (bias current in the case).^41^ Figure 3i shows the NEP of ≈138 pW Hz^−0.5^ at 0.29 THz and 58 pW Hz^−0.5^ at 0.12 THz at nonbiased regime, respectively, superior to other thermal‐based driven detectors in sensitivity and speed.^19^ **Table** **1** summarizes the performance of the fabricated devices with different channel lengths obtained at different titled angles, all of which are characterized under the same conditions. It can be found that the performance is continuing to improve by decreasing the channel length down to 30 nm even though the physical device area is unvaried for all the devices, which is harnessed by the crucial role of the localized field enhancement down to the skin depth limit.

**Table 1:** Comparison of performance for black phosphorus detectors with different channel lengths

θ [°]	*L* [nm]	τ [µs]	Responsivity^a)^ [V W^−1^]		NEP^b)^ [pW Hz^−0.5^]	
			0.12 THz	0.29 THz	0.12 THz	0.29 THz
40	30	0.8	297	135	58	138
60	70	0.8	140	63	121	294
65	100	1.1	48	33	270	440
0	4000	15	5	1.5	4000	12 000

^a)^ *V*_bias_ = 0.2 V

^b)^ *V*_bias_ = 0 V.

To verify further the PTE response in the BP‐based terahertz detector, we carried out additional experiments by probing the PTE effect with infrared‐light pumping. Due to its narrow bandgap, the photon absorption in BP exhibits selective wavelength dependence up to the mid‐infrared wavelength within the single‐particle transition process. The nonequilibrium carriers can be principally excited through either interband or intraband process depending on the incident photon energy. At high photon energy, the carriers are excited to the upper level of conduction band or valence band, and subsequently experience rapid relaxation resulting in the broadening distribution of nonequilibrium hot carriers. Therefore, visible/infrared light pumping could be a viable tool toward further manipulation of the nonequilibrium carrier distribution induced by terahertz waves. As illustrated in **Figure** **4**a, a continuous optical‐pumping with 825 nm light‐beam is uniformly illuminated onto the detector, in order to control the carrier dynamics under the THz field. In the meantime, THz photoresponse shown in Figure 4b decreases when increasing the laser intensity. This phenomenon can be understood as the heating effect of near‐infrared radiation, which eliminates the asymmetric distribution of nonequilibrium carriers and causes photocurrent bleaching. In the meantime, broadening of nonequilibrium carrier distribution and up shift of quasi‐Fermi level result in a smaller Seebeck coefficient difference,^39^, ^47^ reducing further the photosignal (Figure 4c,d). Similar damping trend can also be found with 638 nm visible light as shown in the inset of Figure 4b, corroborating the dominated PTE THz detection mechanism in our detectors. The results are also elaborated with the evolution of current–voltage characteristics under the both the infrared pumping and THz light probing as shown in Section S7 in the Supporting Information.

**Figure 4:**
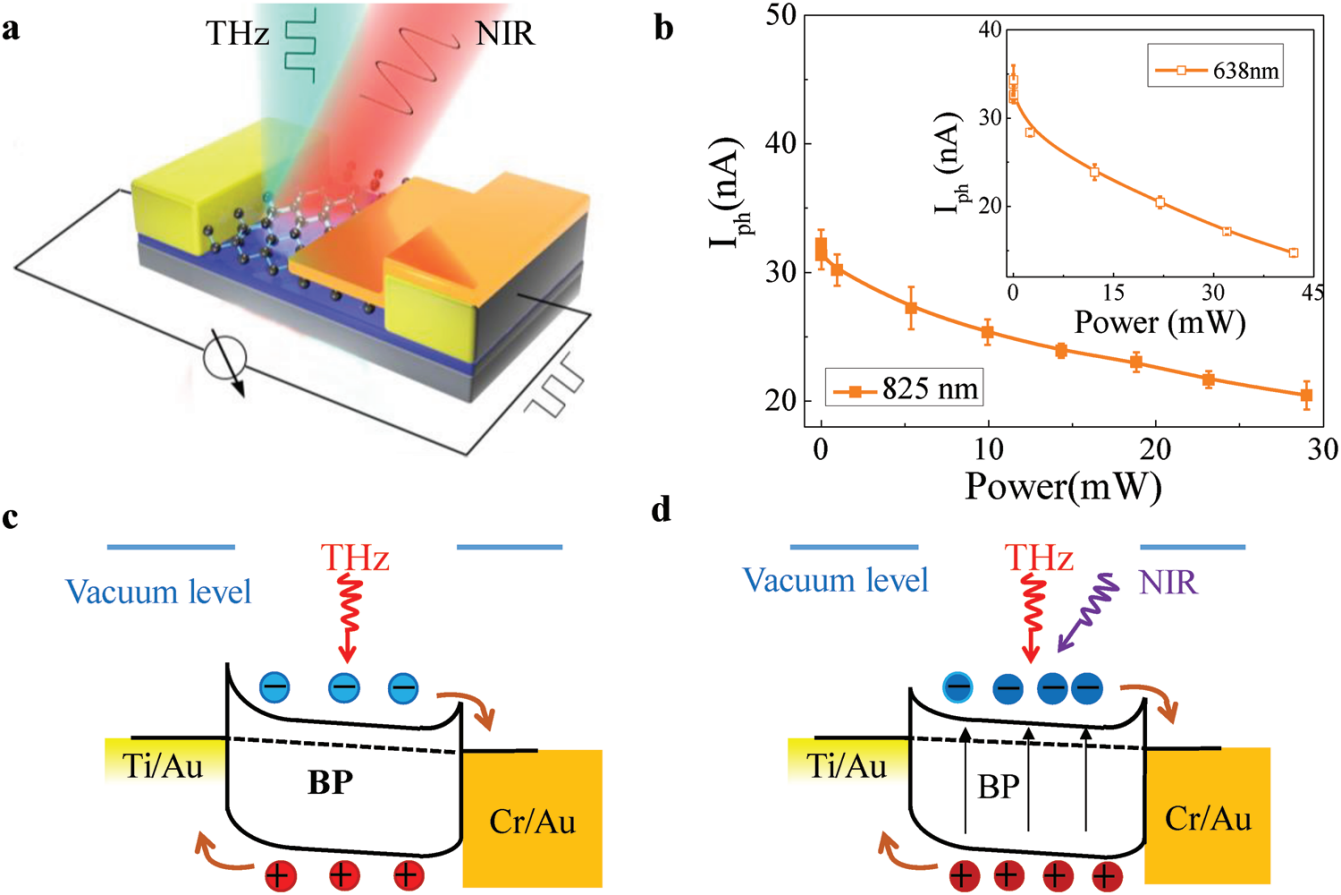
Infrared pumping and probing with THz radiation of the BP in the sub‐100 nm channel detector working in the zero‐biased mode. a) 3D schematics of the device for infrared‐assisted THz photocurrent response. b) THz photocurrent response of the device for varied values of the power of the infrared‐light source. c,d) Optical pumping induced shift of Fermi level eliminates the difference of Seebeck coefficient between the two metal–BP interfaces, behaving like a photoswitch.

Furthermore, a large dynamic range is also confirmed in our PTE detector by using power‐tunable electromagnetic source at 0.04 THz. In this work, we have measured the photocurrent versus output power *P*_out_ as shown in the Supporting Information Section 4, with the power tuned from 0.07 to 30 mW. All the curves are fitted well by a simple power law *I*_ph_ ∝ *P*_in_ *^α^* and α > 0.96 can be retrieved. This phenomenon indicates that the photocurrent depends linearly on the incident power over a large range of more than two orders of magnitude. The reason that the photocurrent depends on the incident power linearly can be attributed to the fact that the photodetector is operated in the weak heating regime, in which ∆*T* << *T*_ambient_, i.e., the change of temperature in the electronic system is much smaller than the ambient temperature *T*_ambient_. When ∆*T* approaches *T*_ambient_, a sub‐linear dependence of photoresponse on the incident power can be expected.^43^, ^48^

To better understand the underlying mechanism of PTE effect dominated photoresponse and important designing rule of thumb, we now discuss more by using a simple analytical model that provides a rationale for our design. It is worth mentioning that BP could be an ideal material to exploit PTE effect for THz detection due to its large Seebeck coefficient and therefore the large thermoelectric figure of merit at the specific crystal orientation.^34^ Besides, PTE effect can also be enhanced if the temperature rise Δ*T* of hot carriers can be predominantly larger than its surroundings. Our design maximizes the Δ*T* rise in a robust way by improving electric field intensity in sub‐100 nm regime, and theoretical simulation indicates that more than three orders of magnitude enhancement of power intensity at the photoactive area. The temperature rise of hot carriers in BP can be simply given by Δ*T* ≈ *P*_abs_/κ, here κ is the thermal conductivity of BP, *P*_abs_ is the absorption power given by *A*_active_ *σE* ^2^/2, and *A*_active_ = *W* × *L* ≈ 0.04 µm^2^ is the active area. The electric field *E* is related with the electric field of incident beam *E*_0_ as *ξE*_0_^2^. Using the same irradiance as in the experiment with the light intensity given by *E*_0_^2^/2*Z*_0_ (*Z*_0_ = 377 Ω is the impedance of free space), the absorption power can be estimated as 9 nW at 0.12 THz and 3 nW at 0.29 THz. In addition, the hot hole gas model of diffusive cooling by electrodes is applicable to make simple estimation of thermal conductivity κ of BP is *κ = LσT*,^9^, ^19^ and *L* is the Lorentz number given by *L = πk*_B_^2^ */*3*e* ^2^, which results in κ = 0.32 nW K^−1^. Following the simulated results, we find the temperature rise of holes can reach over 28 K at 0.12 THz and 3 K at 0.3 THz confirming the weak heating regime.

To be more quantitative, we have measured a photocurrent of *I*_PTE_ ≈ 27 nA at 0.29 THz and 340 nA at 0.12 THz. Taking into account the Seebeck coefficient difference in the range of (*S*_L_ − *S*_R_) ≈ 100 µV K^−1^ for the similar flake (reported experimentally)^49^, ^50^ and measured resistance *R* ≈ 7.3 kΩ, an experimental temperature rise of 2 K for 0.29 THz and 25 K for 0.12 THz can be derived. These values are very close to the retrieved ones on basis of simulated electric field, and the discrepancy is reasonable due to the effect of metallic part and detailed structure around the photoactive area that are probably simplified by simulation, substantiating the role of PTE during the process. Since both the amount of absorption power and the thermal conductivity depend the electrical conductivity, the temperature rise Δ*T* is determined solely by the product of photoactive area and localized electric field in an ideal case. Whereas the localized electric field intensity is negatively correlated with channel length *L*, and thus the sensitivity of a PTE detector should be improved significantly by reducing the channel length,^36^ which is exactly the case of our photodetector. It could be understood more phenomenologically that with the smaller active area, the nonequilibrium hot holes would be expected to arrive at a higher temperature since the power intensity is actually increasing in a smaller scale. Even though we have employed voltage responsivity for better comparison with reported ones, the device is also well suited to the photocurrent mode depending on the subsequent readout electronics. However, there exists significant trade off among speed, responsivity, and photoactive geometry. Typically, the photocurrent mode requires device with smaller resistance and the resultantly faster speed, which can be achieved by simply decreasing the channel length. On the contrary, both the width and length should be reduced simultaneously if the voltage responsivity is needed for electrical readout, and the electrical bandwidth is limited by the large series resistance.

Finally, following the well‐consolidated requirement for THz imaging application, the device is placed at the THz beam focus shown in **Figure** **5**e, in order to confirm reliability and credibility of the PTE detection explored here for addressing application‐oriented issue. The terahertz source is tuned to 0.29 THz and operated at room temperature under ambient environment with 1 kHz repetition rate. As a test object, 2D raster scanning imaging is devoted to single‐pixel detector, with total 200 × 200 points acquired by lock‐in technique, for example, a fresh leaf is glued at focal plane of *x*–*y* axis and the transmitted power can been recorded by 2D translation with the photodetector operating in zero bias and 20 ms integral time, so that the water percentage distribution of the leaf can be revealed. The obtained picture in Figure 5b exhibits high quality that the leaf veins can be clearly visualized, indicating that our PTE detector is already exploitable for large area imaging.

**Figure 5:**
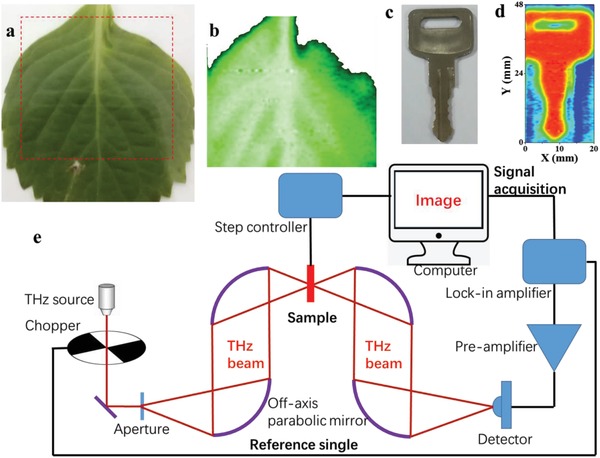
a) The optical picture of a fresh leaf used in the imaging. b) 0.29 THz nondestructive transmission image of the fresh leaf reveals the leaf veins clearly. c) The optical picture of a metallic key. d) THz image for the key enclosed in an envelope. It is clear that our BP‐based terahertz detector allows for inspecting the objects invisible to human eyes. e) Schematic of the setup used for the terahertz detection and imaging of macroscopic objects.

In summary, we propose a novel strategy to improve the performance of THz photodetector by utilization of the photothermoelectric effect in the skin‐depth limit, through ultrashort channel designing down to 30 nm aided by a robust tilted angle metal deposition method. Even without optimization, the devices already exhibit capabilities of high responsivity larger than 297 V W^−1^, noise equivalent power less than 58 pW Hz^−0.5^ as well as fast response across broad photon frequencies, which is superior to other thermal‐based detectors working at room temperature. The PTE effect here is augmented from small photoactive area with large localized electric‐field intensity enhancement beyond the skin‐depth limit as well as the facile fabrication of asymmetric contacts, allowing the improved photon absorption and hot carrier flow without bias. The results are further verified by the quantitative comparison with long‐channel device, which substantiating that the PTE effect is the driving mechanism for the orders of magnitude performance enhancement. Finally, THz imaging experiment is devoted to elucidate the availability of BP‐based PTE photodetector for realistic exploitation in applications of biomedical sensing, nondestructive evaluation and quality control. With such a quantitative understanding, we expect a sufficient room for further sensitivity enhancement through efficient optimization of the resistance‐matched antenna, the resistance, and shape of photoactive area as well as the photoactive materials with appropriate Seebeck coefficient.

## Experimental Section

##### Device Fabrication

Multilayer BP (≈30 nm) was exfoliated, by a blue Nitto tape, onto a substrate of 300 nm SiO_2_ over high resistance Si (*ρ ≈* 20 000 Ω cm). Raman spectroscopy and AFM are used to find multilayer samples and characterize their thickness. “Long” channel (*L* ≈ 4 µm) BP detectors with typical 10 nm/60 nm thick gold (Cr/Au) source and drain contacts were fabricated by sequential ultraviolet lithography patterning, electron beam deposition, and lift‐off processes. Then, a second ultraviolet lithography process was proceeded, opening a window on the channel region of the BP detectors, followed by deposition of 10 nm/60 nm thick Ti/Au film along the titled angle θ. Through controlling the angle, sub‐100 nm channel down to 20 nm can be given rise. Finally, an ALD process was carried out to deposit a 20 nm thick Al_2_O_3_ layer as a protective cover to avoid BP degradation.

##### THz Detection and Imaging

For THz detection, the device is uniformly illuminated with a chopped electronic source and detected the open‐circuit photovoltaic signal or short‐circuit photocurrent signal by using a preamplifier, a lock‐in amplifier, and a high‐speed sampling oscilloscope. The evolution of *I*–*V* curves of the devices was also measured under “ON/OFF” modulated radiation. The frequency was tuned up to 0.12 THz (WR 9.0 Tripler) and 0.24–0.29 THz (WR 2.8 Tripler) output from Virginia Diodes Inc. multiplier connected to a microwave source (Agilent E8257D), the power flux intensity is calibrated by a TK100 power meter. The detector under test was biased and mounted in a focus point of the test system. The whole area of the device S_1_ = 0.2 mm × 0.14 mm = 0.028 mm^2^, which is much smaller than the diffraction‐limited area *S* = λ^2^/4, so the active area is taken to be *S* = λ^2^/4. All the measurements mentioned above were performed under ambient condition at room temperature. During the THz imaging process, the terahertz beam is collimated and focused by four off‐axis parabolic mirrors. The terahertz source is tuned to 0.29 THz in order for reaching better resolution and the whole picture is obtained by 2D scanning of concealed object glued to the translation stage with 20 ms integral time in every step.

## Conflict of Interest

The authors declare no conflict of interest.

## Supporting information

Supporting Information
