# Supplementary material for: Sensitive Terahertz Detection and Imaging Driven by the Photothermoelectric Effect in Ultrashort‐Channel Black Phosphorus Devices
Source: Adv Sci (Weinh). 2020 Jan 19;7(5):1902699. doi: 10.1002/advs.201902699 (PMC7055554; doi:10.1002/advs.201902699)
Supplement: Supplementary file 1 — Supporting Information [file ADVS-7-1902699-s001.pdf]

Copyright WILEY-VCH Verlag GmbH & Co. KGaA, 69469 Weinheim, Germany, 2020.

ADVANCED SCIENCE
Open Access

Supporting Information

for *Adv. Sci.,* DOI: 10.1002/advs.201902699

Sensitive Terahertz Detection and Imaging Driven by the Photothermoelectric Effect in Ultrashort-Channel Black Phosphorus Devices

Wanlong Guo, Zhuo Dong, Yijun Xu, Changlong Liu, Dacheng Wei, Libo Zhang, Xinyao Shi, Cheng Guo, Huang Xu, Gang Chen, Lin Wang, Kai Zhang,* Xiaoshuang Chen,* and Wei Lu*

Supporting Information

Sensitive Terahertz Detection and Imaging Driven by Photothermoelectric Effect in Ultrashort Channel Black Phosphorus Devices

Wanlong Guo[1, 4, 5], Zhuo Dong[2, 6], Yijun Xu[2], Dacheng Wei[3], Libo Zhang[1, 4, 5], Xinyao Shi[2], Cheng Guo[1, 4], Huang Xu[1, 4], Gang Chen[1, 4], Lin Wang*[1, 4], Kai Zhang*[2], Xiaoshuang Chen*[1, 4, 5], and Wei Lu[1, 4, 5]

[1]*State Key Laboratory of Infrared Physics, Shanghai Institute of Technical Physics, Chinese Academy of Sciences, 500 Yu-Tian Road, Shanghai 200083, China.*

[2]*CAS Key Laboratory of Nano-Bio Interface & Key Laboratory of Nanodevices and Applications, i-Lab, Suzhou Institute of Nano-Tech and Nano-Bionics (SINANO), Chinese Academy of Sciences, Ruoshui Road 398, Suzhou, Jiangsu, 215123, China*

[3]*Institute of Molecular Materials and Devices, Department of Material Sciences & Department of Macromolecular Sciences, Fudan University, Shanghai 200433, China.*

[4]*University of Chinese Academy of Sciences, No. 19A Yuquan Road, Beijing 100049, China.*

[5]*School of Physical Science and Technology, ShanghaiTech University, Shanghai 201210, China.*

[6]*School of Nano Technology and Nano Bionics, University of Science and Technology of China, Jinzhai Road 96, Hefei, Anhui, 230026, China*

*Emails: wanglin@mail.sitp.ac.cn;
kzhang2015@sinano.ac.cn;
xschen@mail.sitp.ac.cn;

Section 1 Simulation

The antenna simulation was performed for a frequency range of 0.02–0.5 THz (wavelength of 15 mm–0.6 mm) using FDTD method, a 3D full-wave electromagnetic field solver. The antenna and electrodes were designed in the

simulation to match the actual fabrication of the device.

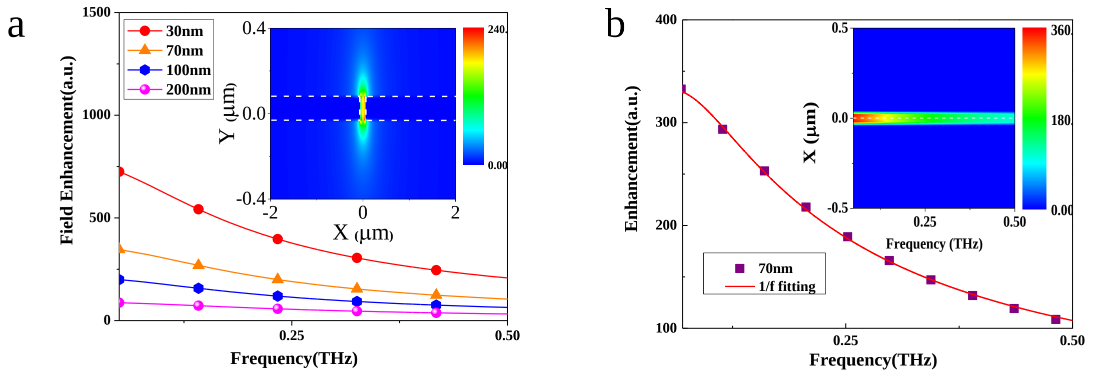

Figure S1. Simulation as the THz wave imping the bow-tie antenna with a small slit in the center. (a) The enhancement of the field at varying frequency from 0.02 THz to 0.5 THz[1].

Section 2 The growth of BP

In this paper, high quality BP crystals were synthesized by an improved mineralizer-assisted chemical vapor transport (CVT) method, using tin tetraiodide (10 mg, Aladdin, 99.99%), tin (20 mg, Aladdin, 99.99%) and red phosphorus (500 mg, Alfa Aesar, 99.999%) as source materials. These initial materials were sequentially transferred into a quartz tube and sealed by a Partulab device (MRVS-1002) under 10^{-3} Pa. The tube was then placed horizontally in the heating zone of a quartz tube furnace with temperature gradient. The representative growth procedure was set as following: First, the furnace was slowly heated from room temperature to 750 °C to prevent explosion due to the excessive pressure inside the tube. After holding at 750 °C for 2 hours, the furnace was then cooled to 500 °C and kept at this temperature for at least 4 hours to promote the crystal growth. Finally, the furnace was cooled down to room temperature with a rate of 1 °C/min. After the growth procedure, large-sized BP crystals with metallic luster could be found at the cold end of the quartz tube.

Section 3 The characterization of BP

AFM measurements were performed by using an Asylum Research Cypher AFM operated at room temperature and ambient conditions. Raman measurements were

performed by using a Horiba Jobin Yvon LabRAM HR 800 system with a 532 nm excitation laser operated at 1 mW, ×100 objective lens with about 1-mm diameter spot size, and 1800 lines per mm grating with about 0.45 cm^{-1} spectral resolution.

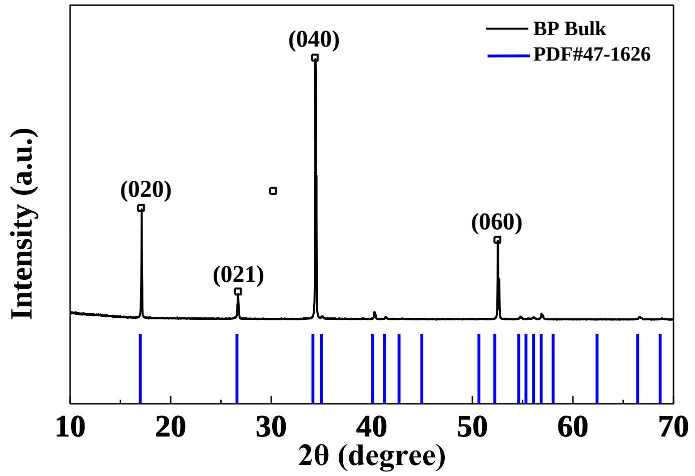

Figure S2. X-ray diffraction pattern of the black phosphorus crystal confirming its high crystallinity.

Section 4 The performance of device

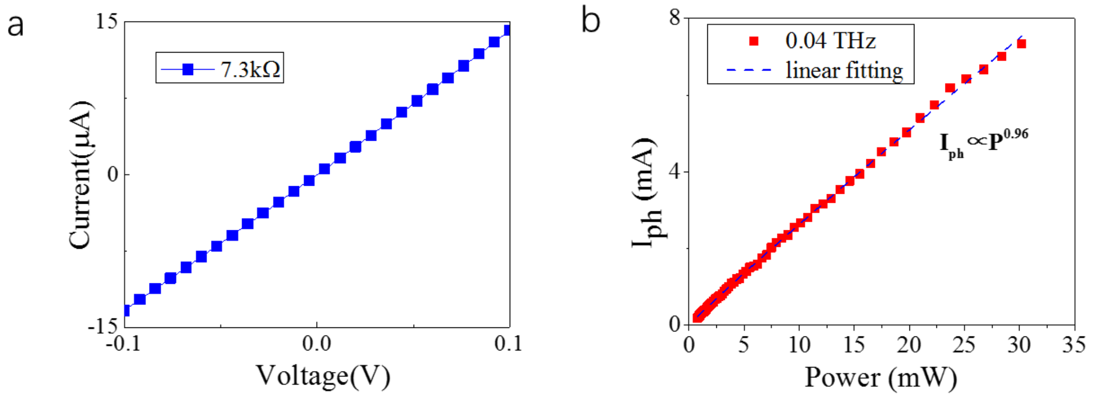

Figure S3 (a) the whole resistance of our device is 7300 Ohm. (b) The measured photocurrent vs. output power P for 0.04T Hz, with the power varied from 0.07 mW to 30 mW. All the curves are fitted well by a simple power law $I_{ph} \propto P^{\alpha}$ and $\alpha > 0.96$ can be retrieved. This phenomenon that the photocurrent depends linearly on the incident power over a large range of more than one order of magnitude indicated that the photodetector is operated in the weak heating regime, in which $\Delta T \ll T_{ambient}$, where ΔT is the change of temperature in the electronic system and $T_{ambient}$ is the ambient temperature[2].

Section 5

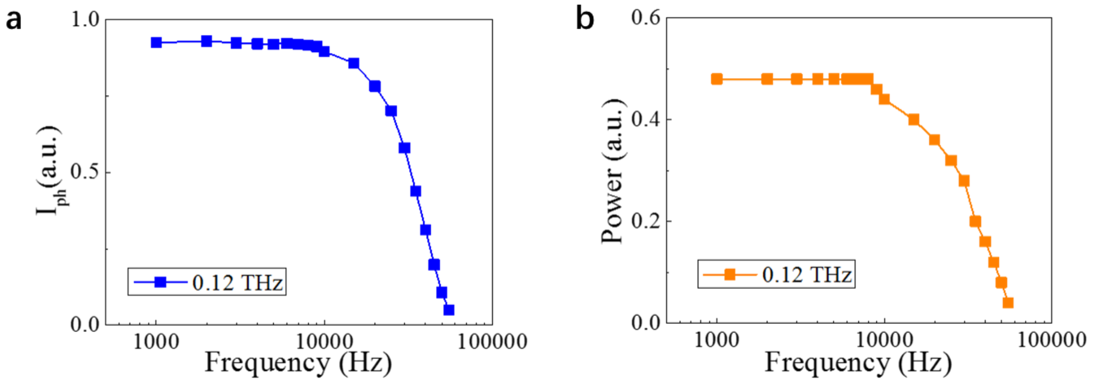

Figure S4 (a) the modulation dependence of photocurrent and (b) the modulation dependence of output power at 0.12THz.

Section 6

Table S1. Comparison of the responsivity and NEP of terahertz detection with reported 2D materials.

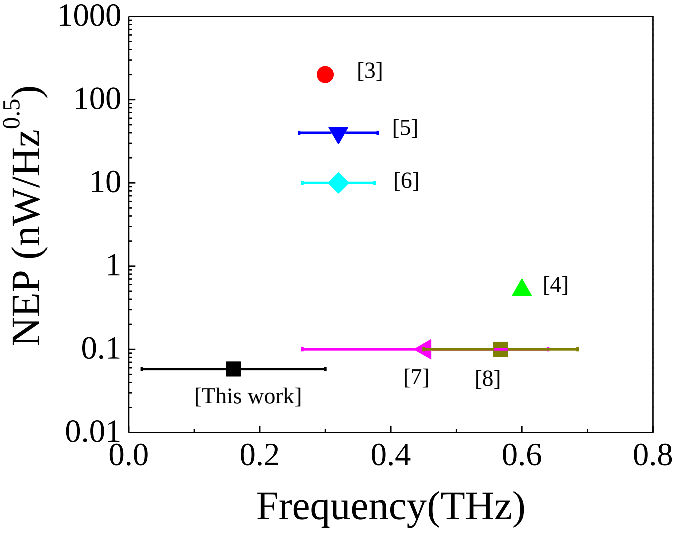

Section 7 I-V characterization with and without THz wave

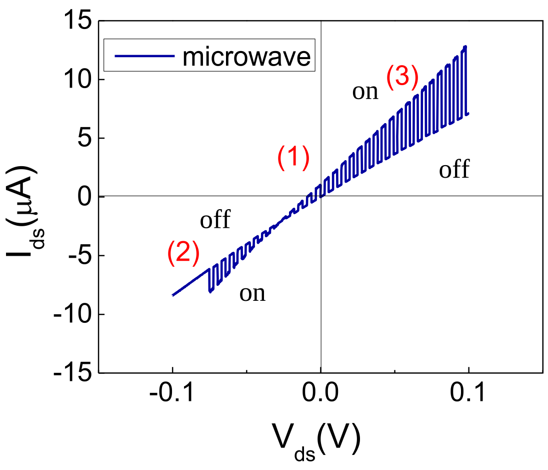

Figure S5a. The evolution of I-V curve for Device A under ON/OFF modulated radiation.

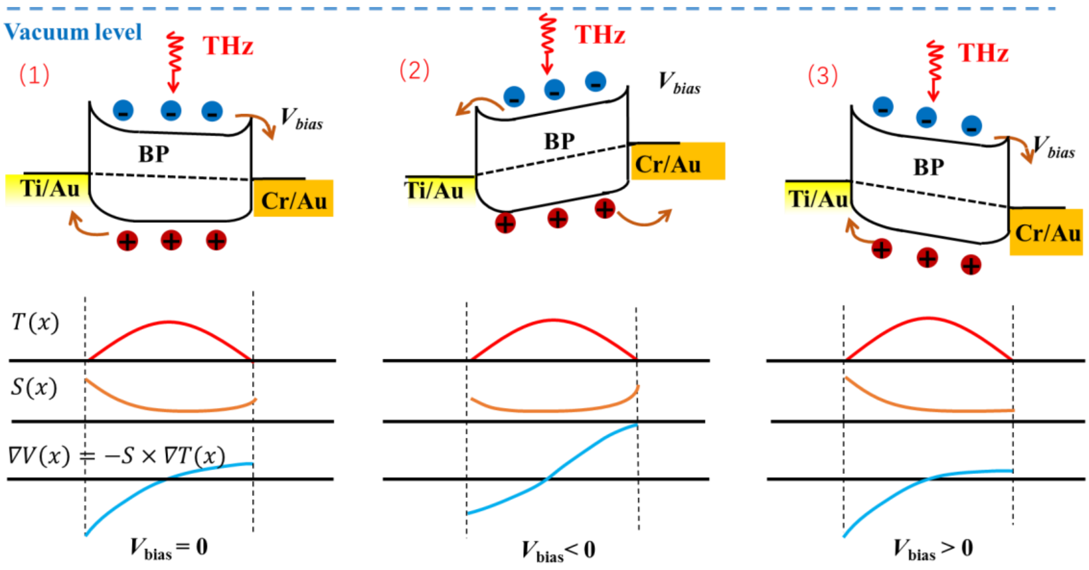

Figure S5b Illustration of the band diagram at the junction regions formed by two dissimilar metallic contacts at varying bias voltage.

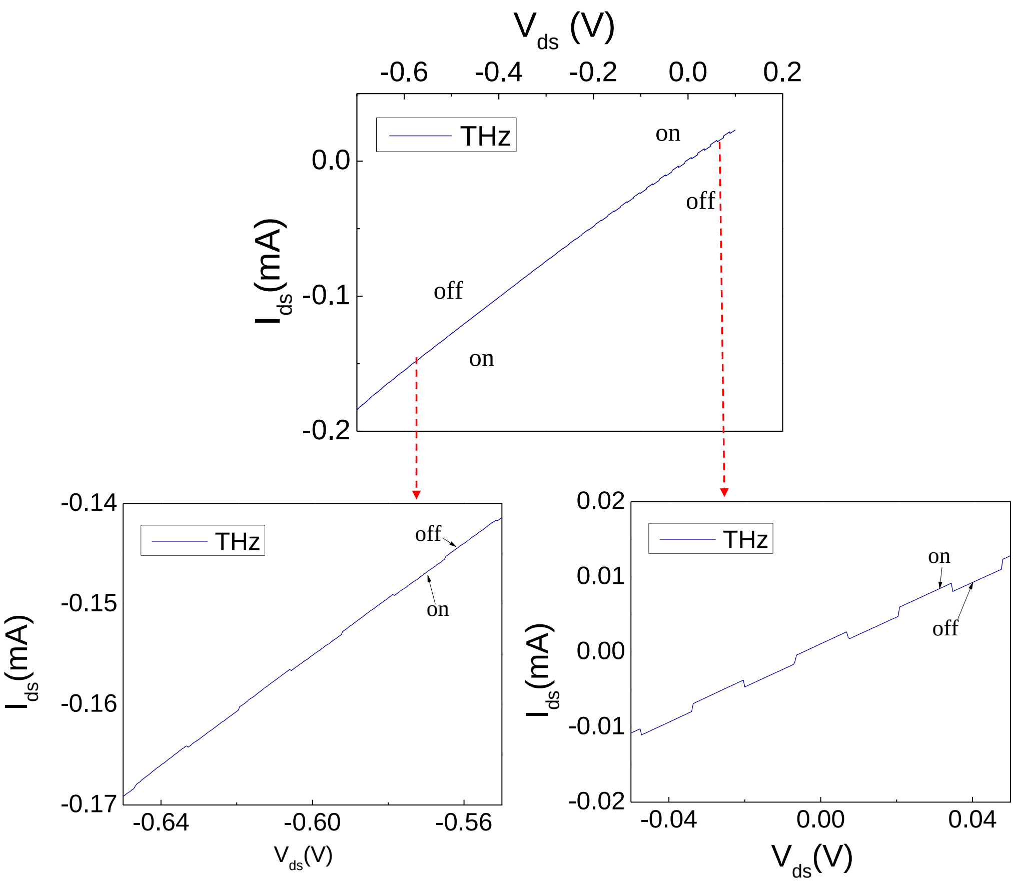

Figure S6. The evolution of I-V curve for Device B under modulated THz radiation.

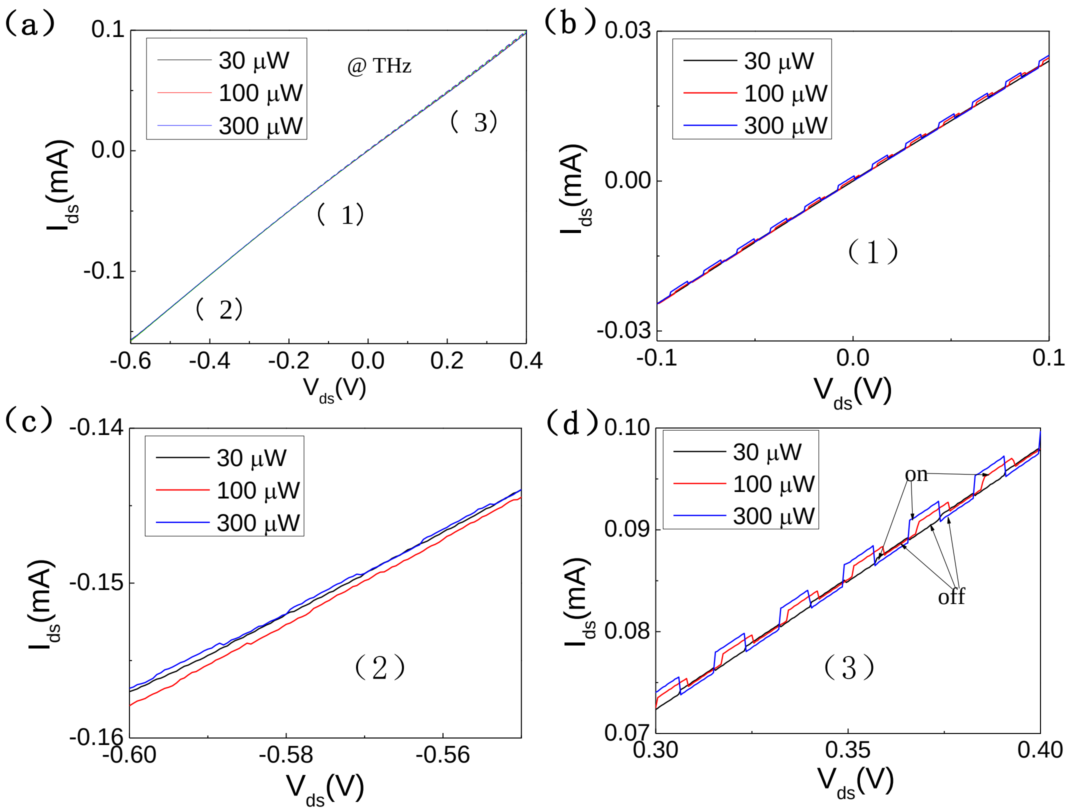

Figure S7 The I-V curve of our detector under modulated THz radiation with varying power. Figure S7b, c and d are the local enlarged view of Figure S7a.

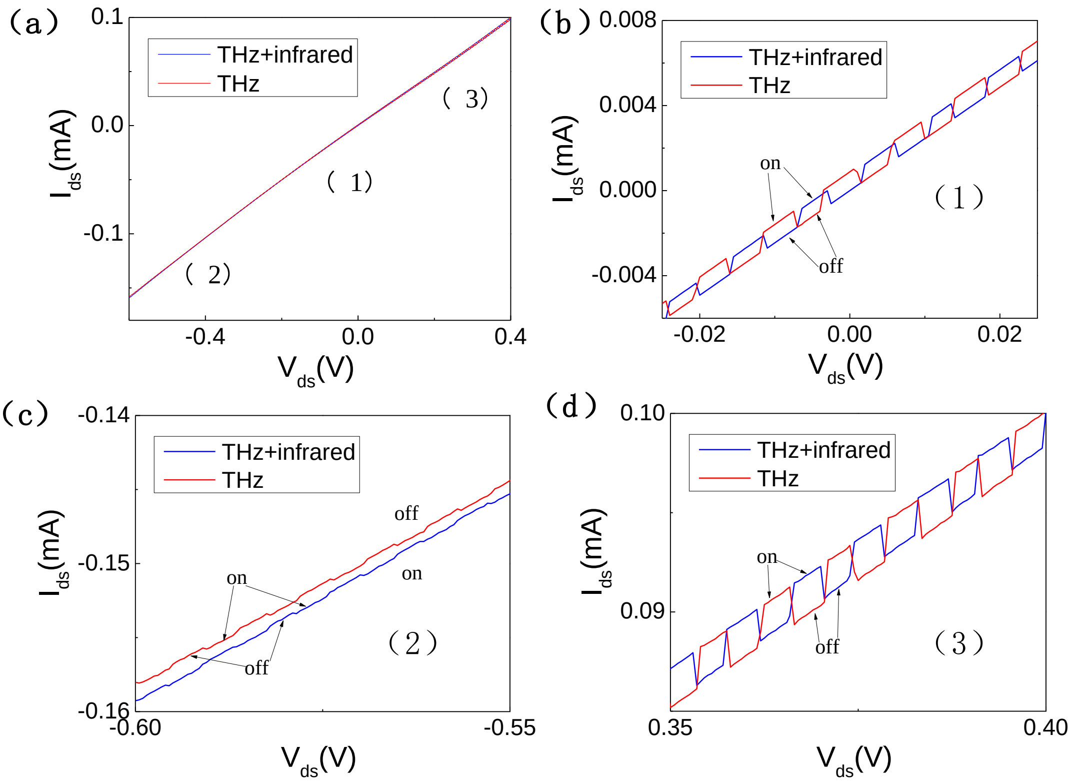

Figure S8 The I-V curves of our device under both modulated THz radiation and 825 nm light-pumping. Figure S8b, c and d are the local enlarged view of Figure S8a. This phenomenon demonstrates that the THz photoresponse decreases when the device is pumped with infrared light.

References

(1). Seo, M. A.; Park, H. R.; Koo, S. M.; Park, D. J.; Kang, J. H.; Suwal, O. K.; Choi, S. S.; Planken, P. C. M.; Park, G. S.; Park, N. K.; Park, Q. H.; Kim, D. S. *Nature Photonics* 2009, 3, (3), 152-156.

(2). Castilla, S.; Terres, B.; Autore, M.; Viti, L.; Li, J.; Nikitin, A. Y.; Vangelidis, I.; Watanabe, K.; Taniguchi, T.; Lidorikis, E.; Vitiello, M. S.; Hillenbrand, R.; Tielrooij, K. J.; Koppens, F. H. L. *Nano Lett* 2019, 19, (5), 2765-2773.

(3). Vicarelli, L.; Vitiello, M. S.; Coquillat, D.; Lombardo, A.; Ferrari, A. C.; Knap, W.; Polini, M.; Pellegrini, V.; Tredicucci, A., Graphene field-effect transistors as room-temperature terahertz detectors. Nat Mater 2012, 11 (10), 865-71.

(4). Tong, J.; Muthee, M.; Chen, S. Y.; Yngvesson, S. K.; Yan, J., Antenna Enhanced Graphene THz Emitter and Detector. Nano Lett 2015, 15 (8), 5295-301.

(5). Viti, L.; Hu, J.; Coquillat, D.; Knap, W.; Tredicucci, A.; Politano, A.; Vitiello, M. S., Black Phosphorus Terahertz Photodetectors. Adv Mater 2015, 27 (37), 5567-72.

(6). Viti, L.; Coquillat, D.; Politano, A.; Kokh, K. A.; Aliev, Z. S.; Babanly, M. B.;

Tereshchenko, O. E.; Knap, W.; Chulkov, E. V.; Vitiello, M. S., Plasma-Wave Terahertz Detection Mediated by Topological Insulators Surface States. Nano Lett 2016, 16 (1), 80-7.
(7). Viti, L.; Hu, J.; Coquillat, D.; Politano, A.; Consejo, C.; Knap, W.; Vitiello, M. S., Heterostructured hBN-BP-hBN Nanodetectors at Terahertz Frequencies. Adv Mater 2016, 28 (34), 7390-6.
(8). Auton, G.; But, D. B.; Zhang, J.; Hill, E.; Coquillat, D.; Consejo, C.; Nouvel, P.; Knap, W.; Varani, L.; Teppe, F.; Torres, J.; Song, A., Terahertz Detection and Imaging Using Graphene Ballistic Rectifiers. Nano Lett 2017, 17 (11), 7015-7020.
